# Antimelanoma and Antityrosinase from *Alpinia galangal* Constituents

**DOI:** 10.1155/2013/186505

**Published:** 2013-08-21

**Authors:** Chih-Yu Lo, Po-Len Liu, Li-Ching Lin, Yen-Ting Chen, You-Cheng Hseu, Zhi-Hong Wen, Hui-Min Wang

**Affiliations:** ^1^Department of Food Science, National Chiayi University, Chiayi 60083, Taiwan; ^2^Department of Respiratory Therapy, College of Medicine, Kaohsiung Medical University, Kaohsiung 80708, Taiwan; ^3^Department of Fragrance and Cosmetic Science, Kaohsiung Medical University, 100 Shih-Chuan 1st Road, San-Ming District, Kaohsiung 80708, Taiwan; ^4^Department of Cosmeceutics, College of Pharmacy, China Medical University, Taichung 40402, Taiwan; ^5^Department of Marine Biotechnology and Resources, National Sun Yat-sen University, Kaohsiung 80424, Taiwan; ^6^Graduate Institute of Natural Products, Kaohsiung Medical University, Kaohsiung 80708, Taiwan

## Abstract

Two compounds, 1,7-bis(4-hydroxyphenyl)-1,4,6-heptatrien-3-one (BHPHTO) and bisdemethoxycurcumin (BDMC) they have been isolated from the rhizomes of *Alpinia galangal*, and the structures of both pure constituents were determined using spectroscopic analyses. The study examined the bioeffectivenesses of the two compounds on the human melanoma A2058 and showed that significantly inhibited the proliferation of melanoma cells in the cell viability assay. This research was also taken on the tests to B16-F10 cell line and showed minor inhibitory consequences of cellular tyrosinase activities and melanin contents. Our results revealed the anticancer effects of *A. galangal* compounds, and therefore, the target compounds could be potentially applied in the therapeutic application and the food industry.

## 1. Introduction

*Alpinia galangal* belongs to the Zingiberaceae family, and the herb grows mainly in South East Asia. It is now cultivated throughout tropical and subtropical Asia, such as India, Egypt, Thailand, Malaysia, Indonesia, and China. The herbs are usually not only used for seasoning but also for traditional medicine. The rhizomes contain essential oil. Many essential oils were obtained by hydrodistillation. The yield of essential oils ranged from 1.32 to 0.143% [1–3]. The wide range of major volatile compounds was identified and characterized by GC and GC/MS as* endo*-fenchyl acetate, zerumbone, 1,8-cineole and myrcene. For *A*.* galangal* rhizome chemical studies, a group of related phenylpropanoids was identified [4, 5]. Among these phenylpropanoids, 1′S-1′-acetoxychavicol acetate (galangal acetate) was the most studied and reported to possess various activities, such as antioxidative, antifungal, antitumor, and anti-inflammatory activities. However, only some studies for *A. galangal* grown in Taiwan were published [6]. Human skin is normally contacted with damage stress, which is produced by external and intrinsic sources, such as ultraviolet (UV) radiation, free radicals, and reactive oxygen species [7]. There are many studies about the skin exposed to oxidative stress or UV radiation and are responsible for aging or tumorigenesis [8]. Melanoma, a malignant tumor of epidermal melanocytes, is one of the most deadly skin cancers. Within the past several decades, the occurrences of cutaneous malignant melanoma have increased because it has a strong propensity to metastasize and, therefore, is one of the most aggressive skin cancers. Unlike other cancers, malignant melanoma is not easy to treat with surgery, radiotherapy, or chemotherapy. A good chemotherapeutic agent will be a naturally occurring agent and can induce cytotoxicity in cancer cells.

In mammals, skin, hair, and eyes, darkening is determined by the synthesis and distribution of melanin. In skin, it is a mixture of pigmented biopolymers that is synthesized in a unique organelle, the melanosome of melanocytes. Excessive biosynthesis of melanin induces various related pigment disorders, such as senile lentigo, melasma, freckles, and pigmented acne scars, that are of particular concern to women as well as men. Their treatment usually involves the use of medicines or medicinal cosmetics containing depigmenting or skin-whitening components. Safe and effective regulators that act to minimize skin pigmentation abnormalities include natural and synthetic depigmenting agents. However, only a few are used as therapeutic agents, primarily because of various safety concerns and low whitening bioactivity. In melanogenesis, L-tyrosine is hydroxylated to dihydroxyphenylalanine (L-DOPA), and then L-DOPA is oxidized to DOPA-quinone with two initial steps [9]. Pigment coloring hair, skin, and eyes because of the key protein, tyrosinase, is recognized to be the first two and rate-limiting enzyme in the biosynthesis of melanins [10]. Recently, much attention has been drawn to the application of tyrosinase inhibitors to medical treatments and cosmetic businesses. Therefore, in clinical usage, tyrosinase inhibitors are being taken for dermatological disorder treatments related to melanin hyperaccumulation and are thus fundamental in cosmetics for depigmentation [11].

## 2. Materials and Methods

### 2.1. Reagents and Materials

All solvents were at analytical grade. Lipophilic Sephadex (LH-20) resin was purchased from Sigma-Aldrich Inc., (St. Louis, MO, USA). Reverse phase (C18; 25–40 *μ*m) sorbent was purchased from Merck Chemical Co. (Darmstadt, Germany).

### 2.2. Extraction and Purification of BHPHTO and BDMC

**Figure 1:**
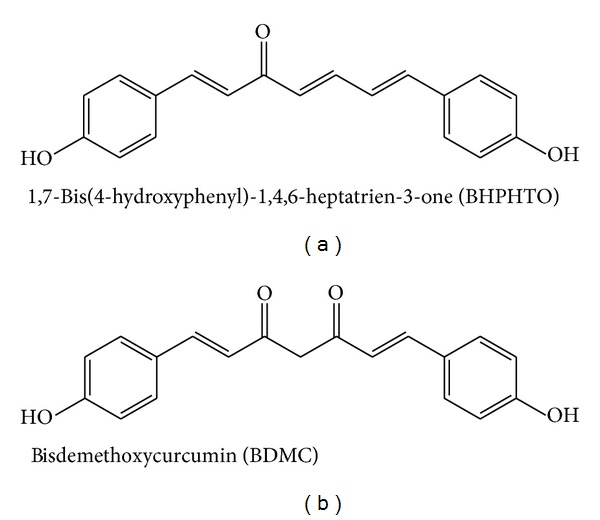
The structure of 1,7-bis(4-hydroxyphenyl)-1,4,6-heptatrien-3-one (BHPHTO) and bisdemethoxycurcumin (BDMC).

The rhizomes of *A. galangal* were collected from the Native Plant Ecological Garden in National Chiayi University. The air-dried rhizomes (300 g) of *A. galangal* were ground into a fine powder and extracted successively with 95% ethanol at room temperature. The crude ethanol extract (CEE) was filtered and evaporated to slurry using a rotary evaporator. The slurry was then suspended with water and partitioned successively three times with hexane (×3) and ethyl acetate (×3). The hexane and ethyl acetate extracts were separately dried using a rotary evaporator at 40°C, followed by freeze-drying for 48 h to give dried hexane and ethyl acetate extract (AG-EtOAC), respectively. The AG-EtOAC fraction was first subjected to passage over LH-20, using 95% ethanol. The eluted solution was collected by test tubes. Only the tubes that contained major components were concentrated and followed by eluting isocratically in the self-pack C18 with 80% methanol solution to obtain BHPHTO and BDMC. The identities of 1,7-bis(4-hydroxyphenyl)-1,4,6-heptatrien-3-one (BHPHTO) and bisdemethoxycurcumin (BDMC) (Figure 1) were confirmed by comparison of their NMR and MS spectral data with those available in the literature [12].

### 2.3. Human Melanoma A2058 Cell and Mouse B16-F10 Melanoma Cell Cultures

The human skin cancer A2058 cell lines were derived and purchased from the Bioresource Collection and Research Center (BCRC number: 60240, Hsinchu, Taiwan, ROC) the American Type Culture Collection (ATCC number: CRL-11147, Manassas, VA, USA). The human metastatic melanoma A2058 cell line is derived from 43-year-old male [13].

And the melanoma B16-F10 cells (BCRC 60031) were obtained from ATCC (Manassas, VA, USA), cultured in DMEM (13.4 mg/mL Dulbecco's modified Eagle's medium, 10 mM HEPES, 143 U/mL benzylpenicillin potassium, 100 mg/mL streptomycin sulfate, and 24 mM NaHCO_3_, pH 7.1) containing 10% FBS, 1% P/S, and incubated at 37°C under 5% CO_2_ atmosphere [14].

### 2.4. Cell Proliferation Assay

The effects of compounds on cell growths were according to the MTT assay procedures [15]. The method is based on the ability of a mitochondrial dehydrogenase from viable cells to cleave the tetrazolium rings of the pale yellow MTT and form impermeable crystals of a dark-blue formazan, thus resulting in accumulation within healthy cells. Briefly, cells were seeded in 96-well plates at a density of 8 × 10^3^ cells/well. The medium was then changed, and cells were maintained in either solvent alone (control cells) or in the presence of the indicated each sample in a final volume of 100 *μ*L in 10% FBS culture medium. Each sample was added to a microplate and incubated under the same conditions as above for 24 h. After 24 h of incubation, the medium was replaced with 100 *μ*L of fresh medium including 0.5 mg/mL MTT. The plate was cultured in a 37°C incubator filled with 5% CO_2_ for 2 h. Each precipitate in a specific dish was dissolved in 100 *μ*L of DMSO to dissolve the purple formazan crystals. After the dishes were gently shaken for 10 min in the dark to ensure maximal dissolution of formazan crystals, the absorbance (*A*) values of the supernatant were measured at 595 nm (UV_vis, BioTek, Winooski, VT). Cell growth was calculated as

(1) Cell  viability(%)=(Asample−A  blank  )(Acontrol−Ablank)×100%.

### 2.5. Measurement of B16-F10 Cellular Tyrosinase Activity

Testing cell tyrosinase activity *in vitro*, B16-F10 melanoma cells (10^5^ cells per well) were placed in 24-well plates in 300 *μ*L of medium containing various concentrations of testing samples and incubated for 2 days [16]. The sample-treated cells were washed with phosphate-buffered saline (PBS) and lysed with 1% Triton X-100/PBS. The enzyme extract of cellular lysate was added to 50 *μ*L of 2 mM L-tyrosine. This reaction was then incubated at 37°C for 3 h in a dark environment, and the absorbance at 490 nm was measured on a spectrophotometer.

### 2.6. Determination of B16-F10 Cellular Melanin Contents

Briefly, we followed the previous method with minor modifications [17]. Cell pellets were dissolved in 2.0 N NaOH containing 10% DMSO and heated at 90°C for 1 h, and suspensions were clarified by centrifugation for 10 min at 10,000 ×g. The amount of melanin was determined spectrophotometrically based on the absorbance at 475 nm [18].

### 2.7. Statistical Analysis

The data were expressed as the mean value obtained in three experiments. Statistical comparisons were performed by Student's *t*-test for paired values.

## 3. Results and Discussion

### 3.1. Cellular Proliferative Depressing Effects of BHPHTO and BDMC on Melanoma Cells

**Figure 2:**
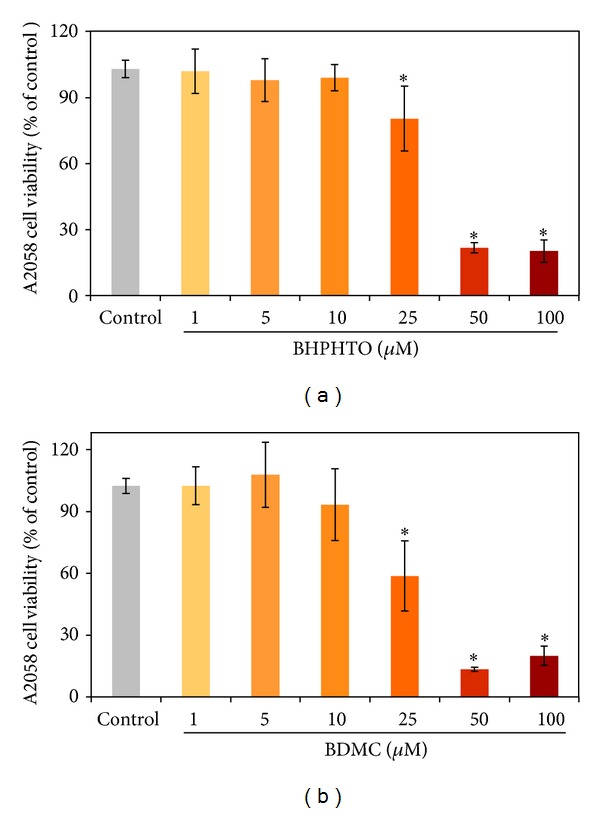
The effects of ginger extracts (BHPHTO and BDMC) on A2058 cell growths measured by MTT assay. The data represented the mean value ± SD of triplicate values for three independent experiments. Comparisons were subjected to Student's *t*-test. Significantly different at *P < 0.05.

The main reason of the death of a patient is due to the tumor proliferation and metastasis [12]. Therefore, it is urgent to find valuable and significant novel biomedicinal components for anticancer therapies. MTT assay illustrated the anticell proliferation of two kinds of human skin melanoma cells, A2058 cells for 24 h treatments. In Figure 2, to evaluate the effects of several ginger (BHPHTO and BDMC) extracts on cancer cytotoxicity, cells were treated with various concentrations from 0 to 100 *μ*M. We demonstrated the cytotoxic abilities on human skin melanoma A2058 cells of the extracts, and cellular proliferations were inhibited in dose-dependent manner when cells were exposed to a high dose of all extracts, BHPHTO and BDMC (50 *μ*M), and cell viabilities exhibited less than 25% after 24 h of treatment.

### 3.2. Cytotoxicity of BHPHTO and BDMC in B16-F10 Cells

**Figure 3:**
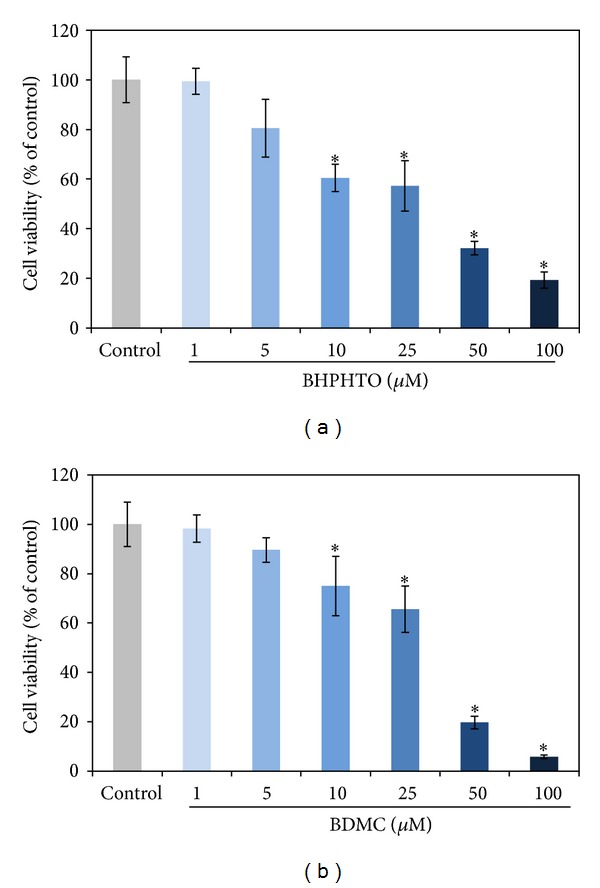
The impact of BHPHTO and BDMC to B16-F10 melanoma cell viabilities. The data represented the mean value ± SD of triplicate values for three independent experiments. Comparisons were subjected to Student's *t*-test. Significantly different at *P < 0.05.

3-(4,5-Dimetylthiazol-2-yl)-2,5-diphenyl tetrazolium bromide (MTT) assay was investigated to study the cytotoxicities of BHPHTO and BDMC in B16F10 cells. The samples were treated with various concentrations, from 1 *μ*M to 100 *μ*M, and the vehicle control group had no testing agents with 0.5% DMSO. As shown in Figure 3. With 25 *μ*M BHPHTO, about 55% B16-F10 cells remained, and with 25 *μ*M BDMC, still 65% B16-F10 cells remained.

### 3.3. B16-F10 Cellular Tyrosinase Activity and Melanin Content of BHPHTO and BDMC

**Figure 4:**
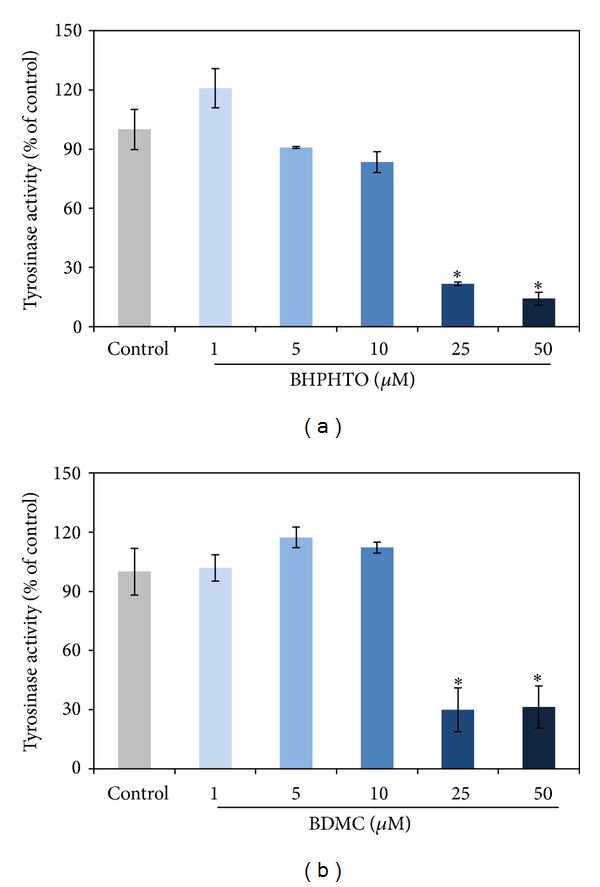
The tyrosinase activity of B16-F10 cells treated with various concentrations of BHPHTO and BDMC. The data represented the mean value ± SD of triplicate values for three independent experiments. Comparisons were subjected to Student's *t*-test. Significantly different at *P < 0.05.

**Figure 5:**
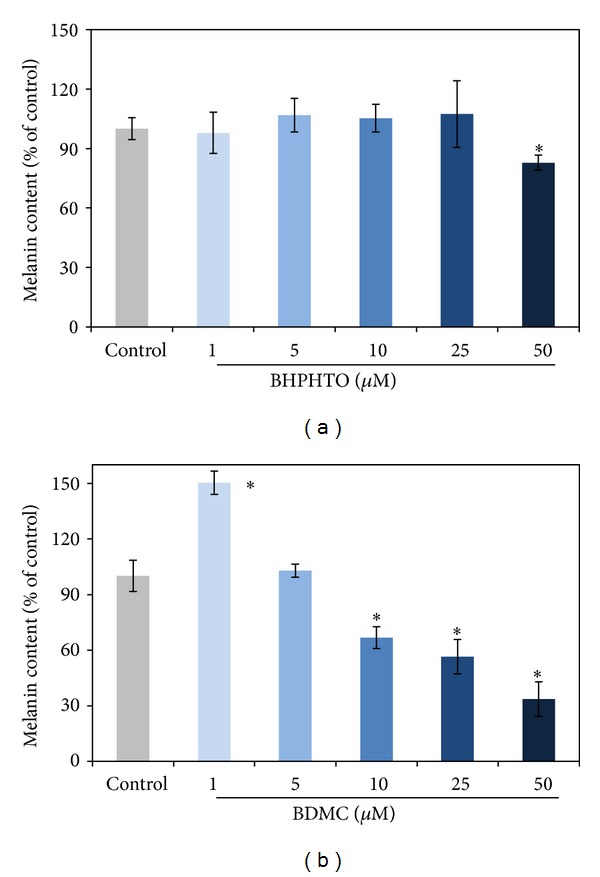
The melanin content of B16-F10 cells treated with various concentrations of BHPHTO and BDMC. The data represented the mean value ± SD of triplicate values for three independent experiments. Comparisons were subjected to Student's *t*-test. Significantly different at *P < 0.05.

Melanin is a vitally important factor in determining the skin color of human. The melanogenesis pathway consists of the enzymatic L-tyrosine hydroxylation and the oxidation of L-dopa to its corresponding dopaquinone [9]. After the two tyrosinase-catalyzed steps, additional multiple biosynthesis steps followed and yielded melanin [10]. We tried to investigate if BHPHTO and BDMC have mouse B16F10 cellular tyrosinase-inhibiting abilities (Figure 4) and melanin content (Figure 5) decreasing power [11]. Although, the tyrosinase activity of B16-F10 results showed that with 5 or 10 *μ*M of BHPHTO and BDMC there is no obvious variation and with concentrations higher than 25 *μ*M tyrosinase activity decreased for low cell viability. But interestingly, even the cell viability is low, the tyrosinase activity is completely high. Melanin content, however, did not show evident reduction and on the contrary, with highest concentrations of BHPHTO and BDMC at 25 *μ*M, the melanin content decreased for low cell viability. As the tendency of tyrosinase activity, melanin content is completely higher than cell viability.
